# A case report on rare co-occurrence of invasive ovarian mucinous adenocarcinoma, unilateral renal agenesis, and bicornuate uterus: is it a new triad?

**DOI:** 10.1186/s12905-024-03130-y

**Published:** 2024-05-20

**Authors:** Orgeness Jasper Mbwambo, Jonaviva Anthony, Alex Mremi, Nicholas B. Ngowi, David H. Mvunta

**Affiliations:** 1 Department of Urology, Kilimanjaro Christian Medical University College (KCMUCO), P. O Box 2240, Moshi, Tanzania; 2 Department of Urology, Kilimanjaro Christian Medical Centre (KCMC), P. O Box 3010, Moshi, Tanzania; 3 Department of Pathology, Kilimanjaro Christian Medical Centre (KCMC), P. O Box 3010, Moshi, Tanzania; 4 Department of Pathology, Kilimanjaro Christian Medical University College (KCMUCO), P. O Box 2240, Moshi, Tanzania; 5 Department of Clinical Oncology, Ocean Road Cancer Institute (ORCI), Barack Obama Drive, P. O Box 3592, Dar es Salaam, Tanzania; 6 Department of Obstetrics and Gynecology, Muhimbili University of Health and Allied Sciences, 9 United Nations Road, Upanga West, P. O Box 65017, Dar es Salaam, Tanzania; 7 Department of Obstetrics and Gynecology, St. Joseph College of Health and Allied Sciences, P. O Box 11007, Dar es Salaam, Tanzania

**Keywords:** Case report, Ovarian mucinous adenocarcinoma, Renal agenesis, Bicornuate uterus

## Abstract

**Background:**

Concomitant invasive ovarian mucinous adenocarcinoma, unilateral renal agenesis and bicornuate uterus is a rare combination. Unilateral renal agenesis has been associated with genital anomalies, such as unicornuate and bicornuate uterus. Furthermore, a wealth of studies has reported the association between unicornuate uterus and ovarian anomalies, such as the absence of an ovary or ectopic ovaries, but rarely has there been a combination of the three to the best of our knowledge. The present case report is the first case presentation with a combination of the three syndromes: ovarian mucinous tumor, unilateral renal agenesis, and bicornuate uterus.

**Case presentation:**

We report the case of a 17-year-old who presented with abdominal distension. On examination, a CT scan revealed a large multicystic abdominal mass on the right side, with an absence of the right kidney while the left kidney was normal in size, appearance, and position. Intraoperatively, massive blood-stained ascitic fluid was evacuated. Additionally, a large whitish polycystic intra-abdominal mass with mucus-like materials and solid areas was attached to the midpoint of the colon and the right ovary, with visible metastasis to the omentum. The uterus was bicornuate. The mass and omentum were taken for histopathology and a diagnosis of invasive ovarian mucinous cystadenocarcinoma with metastasis to the colon and omentum was made after a pathological report.

**Conclusions:**

The presence of these conditions in the same individual could potentially complicate medical management and fertility considerations. Thus, a need for a multidisciplinary medical team, including gynecologists, urologists, and oncologists, to address their unique needs and provide appropriate treatment and guidance. Further research and case studies are needed to better understand the possible association and implications of these rare co-occurring conditions.

## Background

Concomitant invasive ovarian mucinous adenocarcinoma, unilateral renal agenesis, and a bicornuate uterus are a rare combination. Unilateral renal agenesis is estimated to be 4 per 100,000 childbirths [1]. 70–89% of unilateral renal agenesis have been associated with genital anomalies such as unicornuate and bicornuate uterus [2]. Most studies have reported the association between unicornuate uterus and ovarian anomalies, such as the absence of an ovary or ectopic ovaries [2], but rarely has there been a combination of all three conditions to the best of our knowledge.

We report the case of a patient who presented with abdominal pain and a distended abdomen. Intraoperatively it was revealed that the patient had ovarian mucinous adenocarcinoma, which was associated with unilateral renal agenesis and a bicornuate uterus.

## Case report

A 17-year-old female presented to the clinic with abdominal pain that has been ongoing for 3-months. The pain was described as stabbing in nature and worsened when lying flat. Additionally, there was a history of dry cough for 4 weeks and noticeable weight loss. No other abnormalities were noted. There is no history of the same disease among the parents or other family members. During the physical examination, there was evidence of a generalized abdominal distension. A CT scan revealed a large multicystic abdominal mass located on the right side, crossing the midline, with the absence of the right kidney. In contrast, the left kidney was normal in size, appearance, and position.

**Fig. 1:**
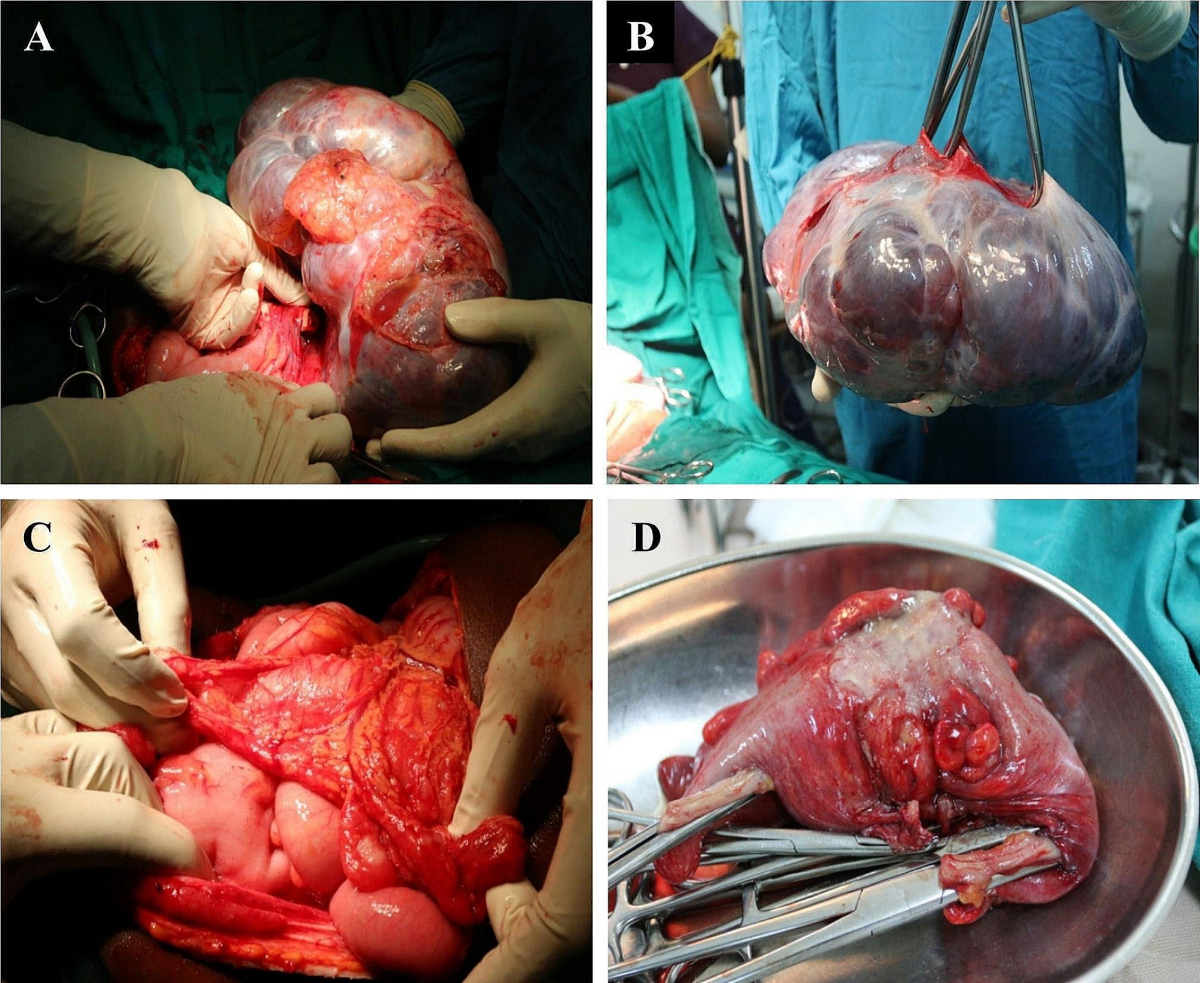
**A** – **D**: Photographs showing ovarian cystadenoma with metastatic deposits. Macroscopic appearance of ovarian adenocarcinoma (**A**). Gross anatomy of the extracted ovarian tumor (**B**). Metastatic involvement of the tumour to the omentum (**C**). Gross image of the resected colon with tumor (**D**)

The patient was subsequently taken to the operating room, where intra-operative findings revealed ascites that were slightly blood-stained. There was also a large polycystic intra-abdominal mass measuring 30 cm x 33 cm x 9 cm. This mass was cystically dilated and consisted of multiple cysts with mucosa material and solid areas (Fig. 1A - B). The whitish tumor was observed at the midportion of the colon (Fig. 1D), partially obstructing the lumen with a thick wall that was presumed to be the ovary, which was attached to the right fallopian tube. No kidney could be palpated on the right side. Multiple metastasis were found on the mesentery (Fig. 1C) and on top of the peritoneum, especially in the suprapubic region. The tumor surrounded the sigmoid colon (Fig. 1D). The uterus was bicornuate, and the left ovary was found to be normal. The mass was then removed and sent to the pathology lab.

**Fig. 2:**
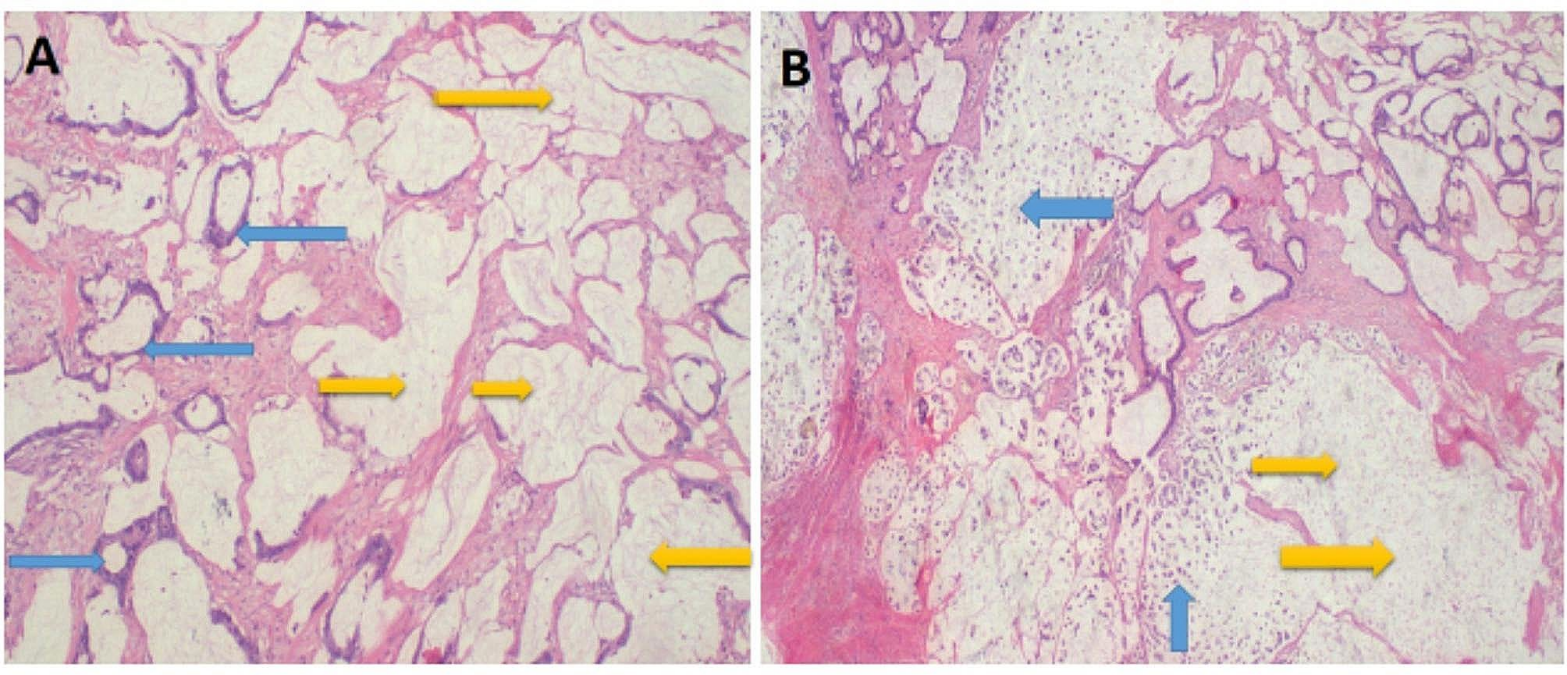
**A** – **B**: Photomicroscopy of the invasive ovarian tumor after H & E staining. Invasive tumor is made up of atypical glandular structures that tend to produce abundant mucin {blue arrows} (**A**). Invasive infiltration of the stromal by single tumor cells or cell clusters {blue arrow} floating in mucin lakes {yellow arrows} (**B**). Magnification;10 x and 4 X of original respectively

**Fig. 3:**
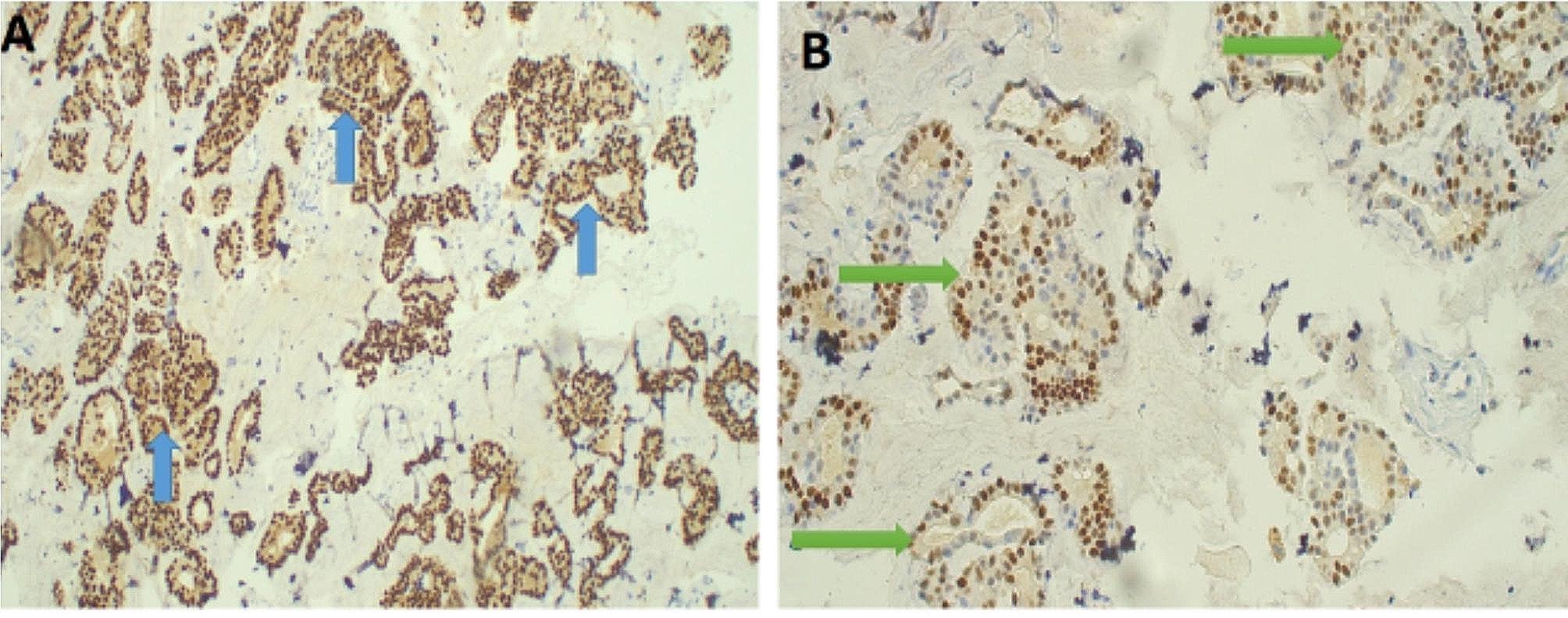
**A** – **B**: Photomicroscopy of ovarian mucinous carcinoma. Highlighting immuno expression of the tumor cells by CK7{blue arrows}; 40 X original magnification (**A**) and Estrogen receptors {green arrows} at 40X original magnification (**B**)

**Fig. 4:**
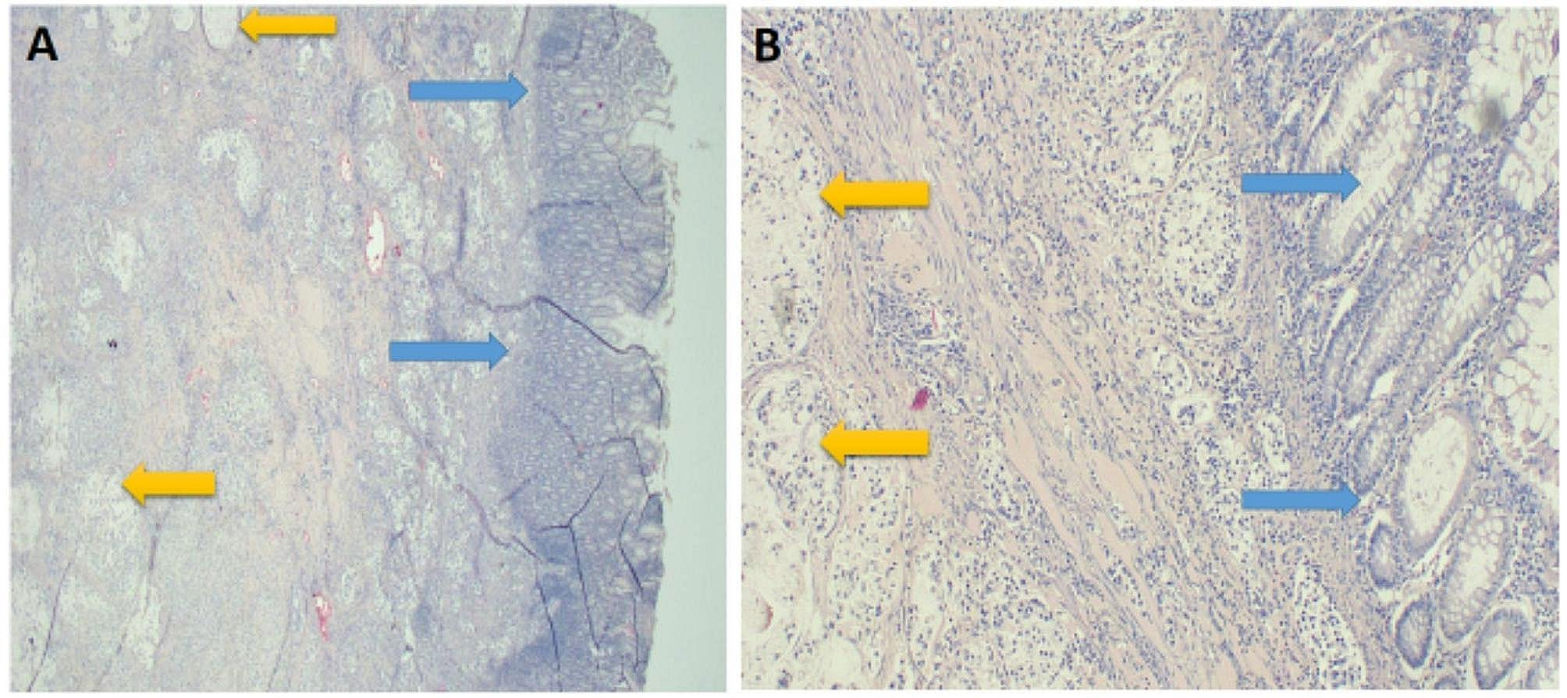
**A** – **B**: Photomicroscopy of colon specimens following H & E staining. Demonstrating normal colonic mucosa {blue arrows} and stroma-based invasive glandular tumor with mucin production {yellow arrows} at 20 x original magnification (**A**), and at 40X original magnification (**B**) respectively

Histology results revealed an ovarian cystic mass showing an infiltrating tumor with glandular differentiation floating in mucin lakes that extended to the sigmoid colon with extensive involvement of peritoneal fat (omentum). A diagnosis of invasive ovarian mucinous cystadenocarcinoma (Fig. 2A – B and 3 A – B ) with metastasis to the colon and omentum was made (Fig. 4A - B).

Post-operatively the patient was then given platinum-based chemotherapy of carboplatin and paclitaxel in the medical oncology department respectively. However, her condition worsened, and she presented with vomiting and loss of appetite. There was a biochemical and clinical progression of the tumor despite receiving chemotherapy. Unfortunately, she passed away after 6 cycles of chemotherapy with elevated tumor markers levels of CA 125 level of 80.600U/ml and carcinoembryonic antigen (CEA) level of > 100.00ng/ml compared to levels of carcinoembryonic antigen of 18.330 ng/mL and CA125 level of 55.4 before chemotherapy.

## Discussion

Unilateral renal agenesis (URA) refers to the absence of renal tissue on one side as a result of the failure of embryonic kidney development [3]. The prevalence of URA has been estimated to be 4/100,000 child birth [1]. Unilateral renal agenesis is thought to occur due to; (1) the failure of the development of mesanephric ducts, and (2) maldevelopment of the ureteral buds, resulting in a lack of induction of the metanephric blastema by the ureteral buds [4].

Unilateral renal agenesis is known to be associated with other genital tract pathologies, such as the absence of vas deferens, unicornuate and bicornuate uterus and skeletal abnormalities, cryptorchidism, and cardiovascular abnormalities [4, 5]. The diagnosis of unilateral agenesis is typically incidental, with hypertrophy of the contralateral kidney. A definitive diagnosis for URA is made by lateral absence of renal tissue on CT or radionuclide scan which is also useful in identifying other congenital anomalies [5].

Ovarian neoplasm in children and adolescents is a rare phenomenon and accounts for 1% of all female genital tract tumors. Surface epithelial tumors account for 20% of the ovarian neoplasms in children [6, 7]. The incidence of epithelial neoplasm has been shown to increase with age among the pediatric age group mostly occurring after menarche [6]. The malignancy rate among epithelial tumors is estimated to be 7.5 to 30%^6^. Most ovarian tumors are asymptomatic at early stages and symptoms initially are not specific then later become more pronounced, which includes a large abdominal swelling which may be associated with dull abdominal pain, sudden loss of weight, and respiratory distress [8]. As in our case, the patient presented with abdominal pain for 3 years and abdominal distension.

Most of the mucinous ovarian tumors are metastatic from the GI tract [9]. In our case, the patient presented with an invasive carcinoma with metastasis in the omentum, mesentery colon, peritoneum, and surrounding part of the sigmoid colon. Ultrasound, CT, and bone scans are used in delineating the tumor’s extent and detecting metastasis in ovarian adenocarcinoma, while most epithelial tumors have no specific tumor marker. However, elevated CA-125 and CEA > 25 have been associated with primary mucinous ovarian adenocarcinoma [9]. In our case, the patient had elevated levels of CA-125 and CEA > 25.

Combining surgery (cytoreductive) and platinum-based chemotherapy is considered the standard treatment for ovarian carcinoma [6, 7]. Mucinous ovarian carcinoma in the early stages of the disease is associated with a good prognosis; however, there is a poor prognosis in advanced stages [9].

A bicornuate uterus refers to a congenital malformation resulting from the failure of fusion of the Mullerian ducts, leading to the formation of a heart-shaped uterus [10]. It is an anomaly that can appear isolated or in combination with other genital anomalies and is estimated to have a prevalence of 0.4% in the general population [10]. Patients usually don’t present any symptoms in adolescence, and diagnosis is typically made through ultrasound and MRI for another condition [11].

However, a bicornuate uterus is associated with various complications, such as pregnancy loss in the first and second trimester, pre-term delivery, malpresentation, and low birth weight babies [12]. The condition is often linked to various renal anomalies due to the close interlinking between the mesonephric and Mullerian ducts during embryogenesis, with the most common anomaly being renal agenesis [13]. In our case, the patient had a bicornuate uterus and unilateral renal agenesis.

The major strength of the present case report is its rare triple occurrence, a novel finding, which may open up new avenues for further research and development in medicine. However, this case report was limited by the lack of genetic testing to confirm its occurrence within the patient’s family.

## Data Availability

The data that support the findings of this case report are not publicly available but can be obtained upon a reasonable request to the corresponding author and with permission from Kilimanjaro Christian Medical Centre (KCMC).

## Abbreviations

CEA: Carcinoembryonic Antigen
KCMC: Kilimanjaro Christian Medical Centre
KCMUCO: Kilimanjaro Christian Medical University College
MUHAS: Muhimbili University of Health and Allied Sciences
ORCI: Ocean Road Cancer Institute
URA: Unilateral renal agenesis

## References

[1] Laurichesse Delmas H, Kohler M, Doray B (2017). Congenital unilateral renal agenesis: prevalence, prenatal diagnosis, associated anomalies. Data from two birth-defect registries: congenital unilateral renal agenesis. Birth Defects Res.

[2] Cerekja A, Dillon KC, Racanska E, Piazze J (2011). Unicornuate uteri associated with contralateral renal agenesis and ovarian anomalies. J Turkish German Gynecol Assoc.

[3] Westland R, Schreuder MF, Ket JCF, Van Wijk JAE (2013). Unilateral renal agenesis: a systematic review on associated anomalies and renal injury. Nephrol Dialysis Transplantation.

[4] Mishra A (2007). Renal agenesis: report of an interesting case. BJR.

[5] Acién P, Acién M (2010). Unilateral renal agenesis and female genital tract pathologies. Acta Obstet Gynecol Scand.

[6] Baert T, Storme N, Van Nieuwenhuysen E (2016). Ovarian cancer in children and adolescents: a rare disease that needs more attention. Maturitas.

[7] Shankar KR, Wakhlu A, Kokai GK, McDowell H, Jones MO (2001). Ovarian adenocarcinoma in premenarchal girls. J Pediatr Surg.

[8] Dutta DC. In: Konar H, editor. DC Dutta’s Textbook of Gynecology: including Contraception. Enlarged&revised reprint of sixth edition. Jaypee Brothers Medical Publishers (P) Ltd; 2014.

[9] Babaier A, Ghatage P (2020). Mucinous cancer of the ovary: overview and current status. Diagnostics.

[10] Chan YY, Jayaprakasan K, Zamora J, Thornton JG, Raine-Fenning N, Coomarasamy A (2011). The prevalence of congenital uterine anomalies in unselected and high-risk populations: a systematic review. Hum Reprod Update.

[11] Letterie GS (2011). Management of congenital uterine abnormalities. Reprod Biomed Online.

[12] Venetis CA, Papadopoulos SP, Campo R, Gordts S, Tarlatzis BC, Grimbizis GF (2014). Clinical implications of congenital uterine anomalies: a meta-analysis of comparative studies. Reprod Biomed Online.

[13] Hall-Craggs MA, Kirkham A, Creighton SM (2013). Renal and urological abnormalities occurring with mullerian anomalies. J Pediatr Urol.

